# PRACTICAL STRATEGIES FOR RETAINING DIRECT CARE WORKERS

**DOI:** 10.1093/geroni/igad104.0975

**Published:** 2023-12-21

**Authors:** Leah Janssen

**Affiliations:** Scripps Gerontology Center / Miami University, Oxford, Ohio, United States

## Abstract

Direct care workers (DCWs), such as state-tested nurse aides, personal care aides, and home care aides are a critical component of the long-term services system. While there is consensus in both the research and practice community on the challenges faced, the plan for a long-term solution continues to be developed. The final paper of this symposium is based on a review of state and national initiatives and interviews with high-performing long-term services and support providers, and culminates into a tip sheet of practical strategies for providers who are seeking to recruit and retain DCWs. The tip sheet is organized in relation to the symposium theme of work culture, which includes elements of creating a welcoming and respectful work environment, employing a participative leadership model, and opportunities for DCWs to anonymously assess work culture. It also discusses staffing ratios that are critical to recruiting and retaining workers, as well as an examination of career ladders, mentorship opportunities, and strengthening training standards. DCW economic well-being (e.g., financial incentives such as wages, bonuses and benefits) and community impact are also explored. The priority of this section is to help providers gain insights on establishing a work culture that is more receptive to DCWs’ needs.

